# Supplementary material for: Saline–Alkaline Stress Resistance of Cabernet Sauvignon Grapes Grafted on Different Rootstocks and Rootstock Combinations
Source: Plants (Basel). 2023 Aug 6;12(15):2881. doi: 10.3390/plants12152881 (PMC10421111; doi:10.3390/plants12152881)
Supplement: Supplementary file 1 [file plants-12-02881-s001.zip › plants-2476401 supplementary.pdf]

Table S1. Parameters and formulae used in JIP-test analysis

Parameters and formulas	Explanation of the parameters
Fo	Minimum fluorescence intensity after dark adaptation
Fj	Fluorescence intensity at J point (2 ms)
Fi	Fluorescence intensity at point I (30 ms)
Fm	Maximum fluorescence intensity after dark adaptation
Fv=Ft-Fo	Variable fluorescence intensity at t
Fv/Fm=(Fm-Fo)/Fm	Maximum photochemical efficiency of PSII
Fv/Fo=(Fm-Fo)/Fo	Potential photochemical efficiency of PSII
Vj=(Fj-Fo)/(Fm-Fo)	Fluorescence intensity at the J step
Vi=(Fi-Fo)/(Fm-Fo)	Fluorescence intensity at the I step
Mo=4(F300μS-Fo)/(Fm-Fo)	Initial slope of the 0JIP fluorescence induction curve
ψo=ETo/TRo=(1-Vj)	The ratio of excitons captured in the reaction center used to push electrons to other electron receptors in the electron transport chain that exceed QA's occupation to push the QA reduced excitons
φEo=ETo/ABS=[1-(Fo/Fm)]×ψo	Quantum yield for electron transport
φPo=TRo/ABS=1-(Fo/Fm)	Primary photochemical reaction of the largest quantum yield
ABS/CSm≈Fm	Light energy absorbed per unit area
TRo/CSm=φPo(ABS/CSm)	Light energy captured per unit area
ETo/CSm=φEo(ABS/CSm)	Quantum yield of electron transport per unit area
DIo/CSm=(ABS/CSm)-(TRo/CSm)	Heat dissipation per unit area
RC/CSm=φPo(Vj/Mo)(ABS/CSm)	The number of reaction centers per unit area
ABS/RC=Mo(1/Vj)(1/φPo)	Light energy absorbed per unit RC
TRo/RC=Mo(1/Vj)	Light energy captured per unit RC
ETo/RC=Mo(1/Vj)ψo	Quantum yield of electron transport per unit RC
PIabs=(RC/ABS)[φPo/(1-φPo][ψo(1-ψo)]	Performance index based on light energy absorption
